# Amodal Volume Completion and the Thin Building Illusion

**DOI:** 10.1177/2041669518781875

**Published:** 2018-06-27

**Authors:** Vebjørn Ekroll, Kathleen Mertens, Johan Wagemans

**Affiliations:** Laboratory of Experimental Psychology, University of Leuven (KU Leuven), Belgium; Department of Psychosocial Science, University of Bergen, Norway; Laboratory of Experimental Psychology, University of Leuven (KU Leuven), Belgium

**Keywords:** amodal volume completion, 3D shape perception, symmetry, perceptual organization, magic, cognitive impenetrability

## Abstract

We report results from an experiment showing that a tall pillar with a triangular base evokes radically different three-dimensional (3D) percepts depending on the vantage point from which it is observed. The base of the pillar is an isosceles right triangle, but the pillar is perceived as just a thin plane when viewed from some vantage points. Viewed from other vantage points, the perceived 3D shape of the pillar corresponds to a square or rectangular base. In general, our results suggest that the visual system uses a preference for rectangularity (or symmetry) to determine the 3D shape of objects. The amodal impressions of the invisible backside of the pillar are often quite compelling, and the corresponding illusions persist even when the observers know the true shape of the pillar. Interestingly, though, the compellingness and definiteness of the amodal impression of the pillar’s backside depends on the vantage point. This is reflected in corresponding differences in the interobserver variability of the 3D shape judgments. We also discuss how variants of this illusion are used as a powerful tool in the art of magic.

## Introduction

When we look at a real-world scene, visual input at the retina is produced only by those parts of the objects in the scene that happen to (a) face toward us and (b) not be occluded by other objects in the foreground. These visible parts of the objects in a visual scene can be described as an empty façade of two-dimensional surfaces facing the observer, in much the same way as a face mask can correspond to all the visible parts of a human head as viewed from a certain vantage point. Intuitively, it would seem natural to assume that a mental representation of this façade—such as Marr’s famous 2 1/2-D sketch—defines where visual perception ends and cognition begins (Marr, 1982, p. 268), because only points on this façade produce visual input.

**Figure 1.**
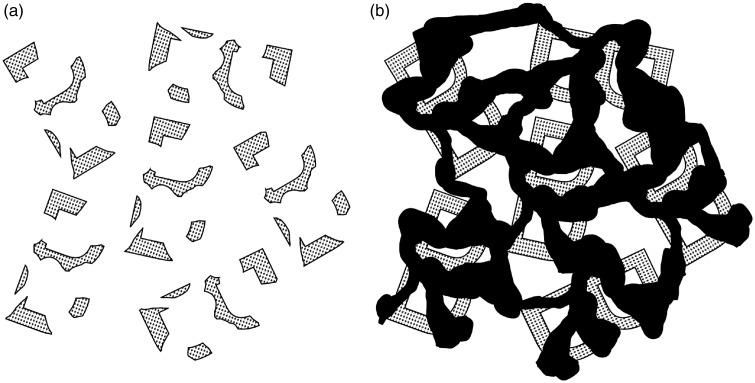
The Bregman illusion. The gray fragments shown in (a) and (b) are identical. In (b), the fragments are easily and effortlessly recognized as the directly visible parts of partially occluded but complete B’s. This demonstrates how unconscious perceptual mechanisms analyzing occlusion relationships aid object recognition. Adapted from Bregman (1981).

Research on amodal completion (Kanizsa, 1985; Michotte, Thinès, & Crabbé, 1964; van Lier & Gerbino, 2015), however, indicates that our mental representations of the occluded parts of a visual scene often have surprisingly much in common with ordinary visual representations. First, as illustrated in Figure 1, our experience of the hidden parts of an object often involves a curious and compelling sense of “perceptual presence” (Leddington, 2009) or “amodal presence” (Michotte et al., 1964). Second, as also illustrated by the same demonstration, the processes underlying our experience of the occluded parts often seem to operate in an automatic and subjectively effortless way. Third, our experience of the occluded parts of objects often has functional influences on other aspects of perceptual processing such as the perception of motion (Holcombe, 2003; Kim, Feldman, & Singh, 2012; McDermott, Weiss, & Adelson, 2001; Scherzer & Ekroll, 2009, 2012; Shimojo & Nakayama, 1990), object recognition (Johnson & Olshausen, 2005), transparency perception (Anderson & Schmid, 2012), and even the felt size of the observer’s own body parts (Ekroll, Sayim, Van der Hallen, & Wagemans, 2016). Fourth, our experience of the hidden parts of objects is often impervious to conscious knowledge or reasoning (Gerbino & Zabai, 2003; Kanizsa, 1985; Michotte et al., 1964; Nielsen, 2008). For instance, a semispherical shell viewed from the convex side will continue to look like a complete ball even when the observer knows that it is just a hollow shell (Ekroll, Sayim, & Wagemans, 2013; Ekroll et al., 2016). This kind of cognitive impenetrability is often considered to be a hallmark of perceptual processes at large (Firestone & Scholl, 2015; Leslie, 1988; Pylyshyn, 1999).

**Figure 2.**
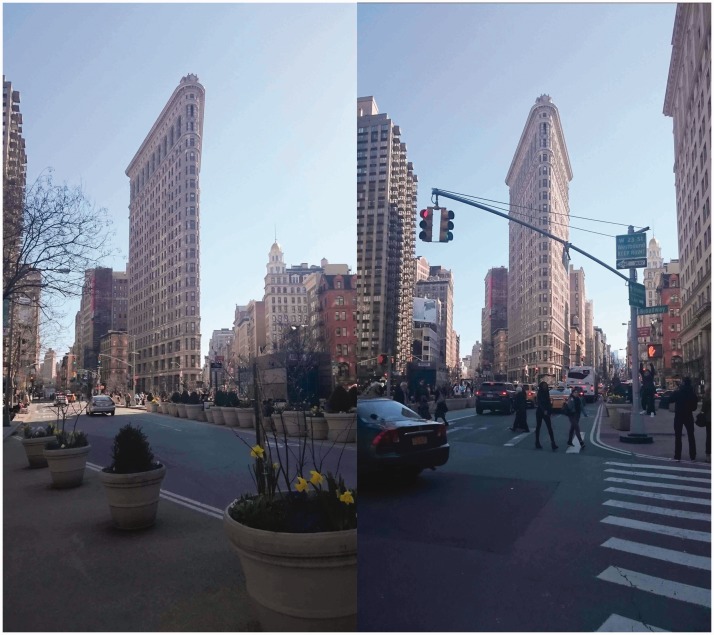
Two pictures of the Flatiron building in New York photographed from slightly different vantage points. Note how the building looks much thinner in the picture on the left.

**Figure 3.**
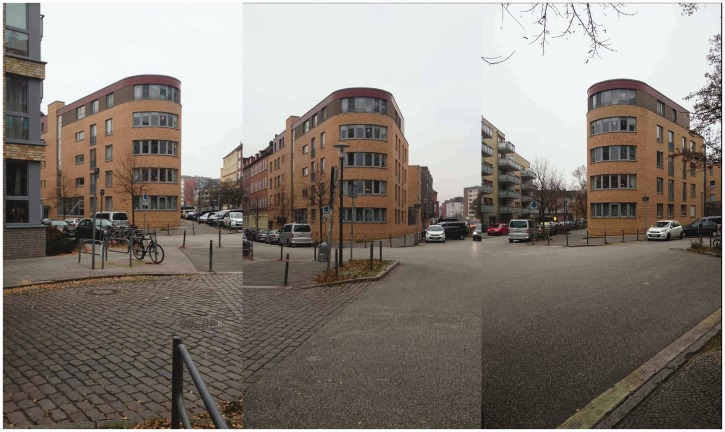
Three pictures of the same building taken from three different vantage points. Note how the building looks much thinner in the left and right photos, as if the hidden long sides were running parallel to the visible long sides.

The present research was motivated by the observation that some buildings (Nielsen, 2008, see his Figure 1(g)) and some other objects such as tables used by magicians (Sharpe, 1992), sometimes look quite a lot thinner than they actually are. Figure 2 illustrates this effect as it occurs by viewing the Flatiron building from two slightly different viewing positions. The building looks thinner in the photo on the left, as if the hidden long backside of the building was running approximately parallel to the visible long side. Figure 3 shows another, perhaps even more striking version of this illusion.

These illusions are presumably best described as cases of amodal volume completion (Tse, 1999a; van Lier, 1999; van Lier & Wagemans, 1999) because they refer to the perceived volume of the hidden backside of things, but unlike most known demonstrations of amodal volume completion, the perceived volume is *less* than the actual (and known) volume.

**Figure 4.**
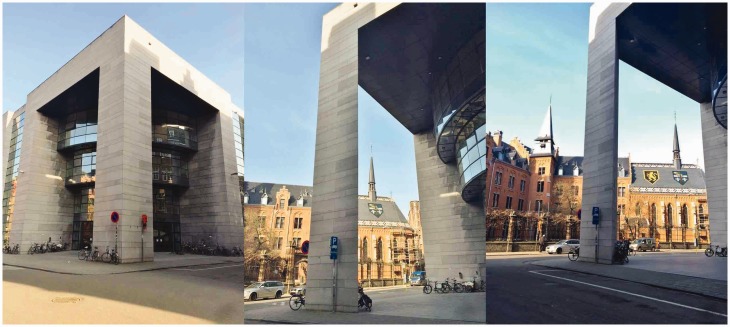
Photos of the pillar used in our experiment taken from three different viewing positions (from left to right: Positions 1, 7, and 9 as indicated in Figure 5). Although the pillar might appear to have a square horizontal cross section in the left photograph or be thin like a piece of cardboard in the middle photograph, the cross section is actually a isosceles right triangle, as shown in Figure 5.

**Figure 5.**
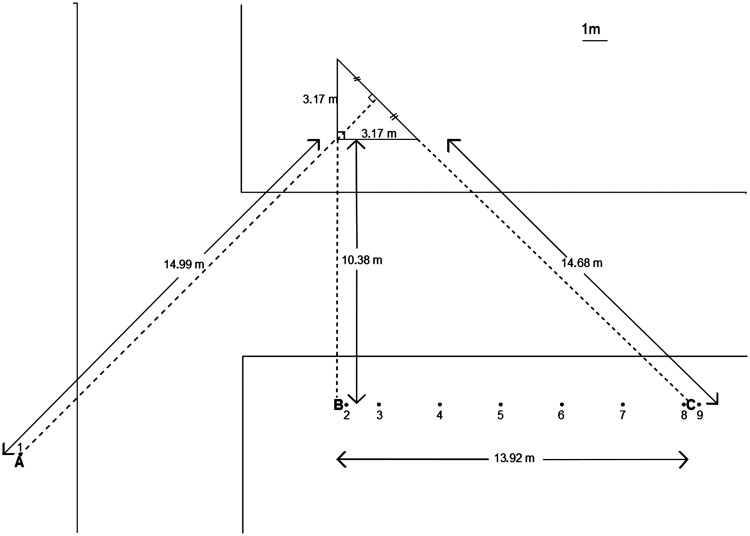
Ground plan showing the triangular pillar and the positions (1–9) from which the observers viewed it. The pillar was located at the intersection of two streets, which are schematically indicated in the drawing. All of the viewing positions (1–9) were located on the pavement. As explained in the text, the location of the viewing positions is most easily described relative to the Reference Points A, B, and C.

In our experiments, we investigated a particularly striking version of this illusion evoked by a tall vertical pillar with a horizontal cross section corresponding to an isosceles right triangle (see Figures 4 and 5). To anticipate, our results show that the perceived three-dimensional (3D) shape of the pillar depends strongly on the viewing position and departs radically from the true triangular cross section of the pillar even though this is known to the observers. Importantly, the perceived 3D shape corresponds to a cross section that can be described as rectangular (a square, a rectangle, or a thin rectangle/line) in almost all cases. A further interesting observation is that the immediate amodal impression of the pillar’s backside seems to be more compelling and definite when viewed from some vantage points than when viewed from others. This is also reflected in the variability of the observers’ 3D shape judgments. In the last section of the article, we shall also discuss how variants of this illusion are used as a powerful tool in stage magic.

## Experiment

### Methods and Procedure

In the experiment, we asked observers to look at a tall^1^ vertical pillar with a triangular cross section belonging to a building in the city of Leuven^2^ and to report how they experienced the backside of it by drawing the missing pieces of a ground plan showing their position and the visible sides of the pillar. Figure 4 shows the pillar, and Figure 5 shows a ground plane illustrating the nine different positions from which the observers looked at the pillar. The cross section of the pillar was an isosceles right triangle with the measures given in Figure 5.

#### Viewing positions

The nine viewing positions used are most easily defined in relation to three Reference Points A, B, and C (Figure 5). Eight of the nine viewing positions used were distributed on a line BC parallel to one of the short sides of the triangle. The Reference Point B was collinear with one of the short sides of the triangle, and the point C was collinear with the long side of the triangle. We also used an additional viewing position located at the Reference Point A. From here, the observers had a symmetrical view of the two short sides of the triangle. That is, the Viewing Position 1 was located on the symmetry axis of the triangle. To ensure that the observers could stand securely on the sidewalk rather than on the street, we had to locate this viewing position a bit further away from the triangle than the other viewing positions (which were also located on the sidewalk). For ease of reference, we number the viewing positions from 1 to 9 according to their horizontal position from left to right in the ground plan shown in Figure 5. Viewing Position 1 coincided with Reference Point A. Viewing Position 2 was located 30 cm to the right of Reference Point B, making it impossible to see the second short side of the triangle. Viewing Position 3 was chosen such that the observer had a symmetrical view of the short side of the triangle. That is, the Viewing Position 3 was 158.5 cm (half the width of the short side of the rectangle) to the right of Reference Point B. Viewing Position 9 was 30 cm to the right of Reference Point C such that the observer could just see the long side of the triangle. Viewing Position 8 was only 60 cm to the left of Position 9, but because it was 30 cm to the left of Reference Point C, the long side of the triangle was no longer visible. The remaining viewing positions (4, 5, 6, and 7) were distributed in equal steps between Positions 3 and 8. Thus, from Viewing Position 3 to Viewing Position 8, the spacing between neighboring viewing positions was 220.7 cm.

#### Task and procedure

**Figure 6.**
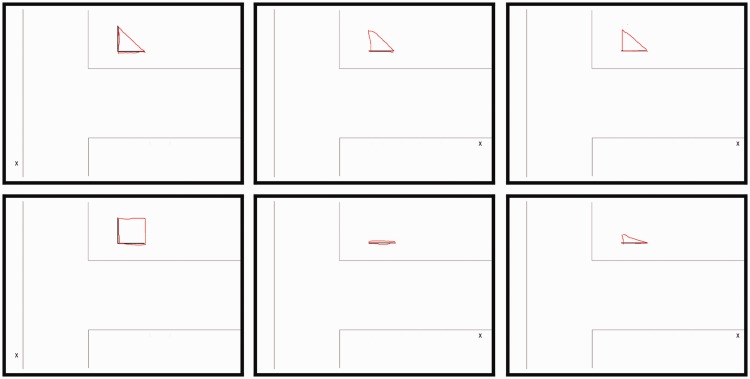
A few selected examples of the drawings made by one of the observers in our experiment. The observers drew their impression of the pillar in red on top of a ground plan showing their own current viewing position as a small x. The ground plan also showed the currently visible short side(s) of the triangular pillar. The drawings in the left row were made at Viewing Position 1, those in the middle row at Viewing Position 8, and those in the right row at Viewing Position 9. The ones in the top row were drawn according to the “cognitive criterion,” and those in the bottom row were drawn according to the “perceptual criterion.”

In a given trial, the observer was positioned at one of the nine viewing positions and asked to indicate their experience of the pillar by completing a ground plan drawing showing their own current position and the currently visible short side(s) of the pillar (see Figure 6). The drawing was presented on the screen of a laptop computer, and the observers used the mouse of the computer to draw the perceived shape of the pillar. They were asked to always draw a completely closed curve (rendered in red) on top of the ground plan drawing (rendered in black). That is, they should not only draw the occluded parts of the pillar but also include the visible parts already shown in black in the ground plan drawing. This obviously produces redundant information but had the advantage that it greatly facilitated subsequent automated data analysis (see Appendix) of the completed drawing.

Each observer was asked to indicate their experience according to two different criteria, namely to draw the shape of the pillar according to their knowledge of it (“cognitive criterion”) and to draw it according to their immediate perceptual experience of it (“perceptual criterion”). To ensure that all the observers had the same knowledge of the pillar prior to data collection, they were asked to walk around the pillar and look at it from all sides before data collection commenced. Accordingly, collecting data using the cognitive criterion might seem unnecessary because the result is not likely to be very surprising. However, the data collection using the cognitive criterion, which was performed prior to the data collection using the perceptual criterion, actually served two important purposes. First, the data obtained using the cognitive criterion provide a baseline estimate of how much the observers’ drawings can be expected to deviate from ground truth due to inaccuracies inherent to the act of drawing itself (eye–hand coordination inaccuracies, problems related to drawing with the computer mouse, etc.). Second, and more important, we believe that using these two different criteria makes it easier and more natural, particularly for the naive and untrained psychophysical observers participating in this experiment, to understand and comply with the instruction to report their immediate perceptual experience (Ekroll et al., 2016).

Immediately prior to the experiment, we used a demonstration of the amodal volume completion of a semispherical shell into a complete ball (Ekroll et al., 2013, 2016) to illustrate to the observers how the perceptual experience of the shape of an object may in some cases differ from its known shape. We started by putting a small semispherical shell on a table in front of the observers. At this point, it is obvious that the object both is and looks like a semisphere (as all observers confirmed), but when the object is lifted off the table keeping the convex side oriented toward the observers eye, it immediately starts looking like a complete ball (as all observers confirmed), despite the explicit knowledge that it is just a semisphere (Ekroll et al., 2013).

To control for potential effects of the sequence in which the observations at the different viewing positions were made, the 30 observers participating in the study were divided into three equal groups. One group of observers visited the viewing positions in the order 1 to 9, the second group visited them in the reverse order (9–1), and the third group visited them in the order 8 to 1, then 9.

The experimenter accompanied the observers through the nine viewing positions (which were marked by crosses on the pavement) informing them about the current task, holding the laptop and saving the responses. Each experimental session lasted about 30 minutes.

#### Participants and ethical approval

Fourteen psychology students from the University of Leuven participated in exchange for course credit. All methods and procedures were approved by the Ethical Committee of the Faculty of Psychology and Educational Sciences at KU Leuven, and written informed consent was obtained prior to the experiments.

### Results

Figure 6 shows a few selected examples of the drawings made by the observers in our experiment. Such raw drawings formed the basis for all subsequent analyses.

#### Qualitative analysis

**Figure 7.**
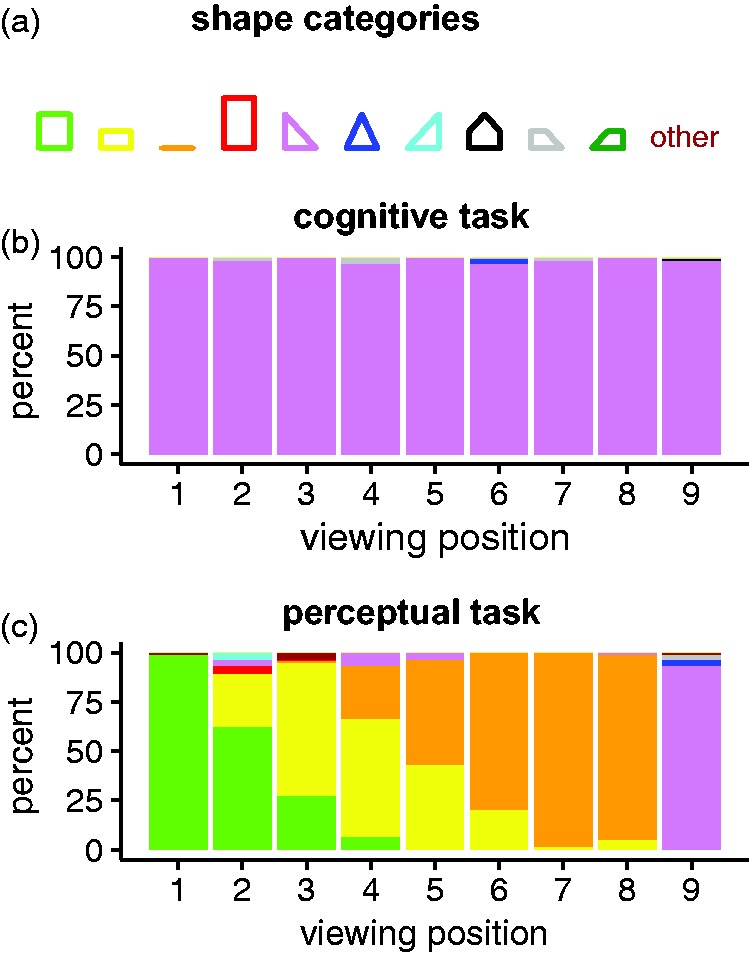
Four raters categorized each of the individual drawings made by the observers into one of the 11 shape categories shown in (a). The data shown in (b) and (c) are percentages of each shape category pooled across all four raters. Irrespective of viewing position, almost all of the drawings made by the observers according to the cognitive criterion (b) were categorized in agreement with the true shape of the pillar (a rightward-pointing isosceles right triangle). The shapes drawn according to the perceptual criterion (b), however, were mainly rectangular (square, rectangle, or thin line) at Viewing Positions 1 to 8 and mostly similar to the true shape of the pillar at Viewing Position 9.

To gain an overview of what kind of shapes the observers drew in the experiment, we asked four raters (authors K. M. and V. E., and two additional independent observers) to categorize each of the original drawings, using the 11 shape categories shown in Figure 7(a). The raters were instructed to regard the shapes in Figure 7(a) as prototypes of more general shape categories allowing for quantitative changes in absolute size and aspect ratio. Unsurprisingly, the drawings made by the observers according to the cognitive criterion were categorized as having the true triangular shape of the pillar in almost every single case (Figure 7(b)). For the perceptual task, on the other hand, the kinds of shapes drawn by the observers depended strongly on viewing position (Figure 7(c)). The kind of shapes drawn in the large majority of cases at Viewing Positions 1 through 8 can all be described as rectangles (in the broad sense, including the square and the thin horizontal line). As the viewing positions move away from 1, where almost all of the drawings were categorized as squares, we first see a gradual change toward a dominance of flat horizontal shapes and subsequently a change to a clear dominance of drawings categorized as a thin horizontal line at Viewing Positions 7 and 8. At Viewing Position 9, however, the shape category corresponding to the true triangular shape of the pillar is clearly dominant. On the whole, shape categories other than the leftmost three and the fifth from left in Figure 7(a) were used very infrequently or not at all.

#### Quantitative analysis

**Figure 8.**
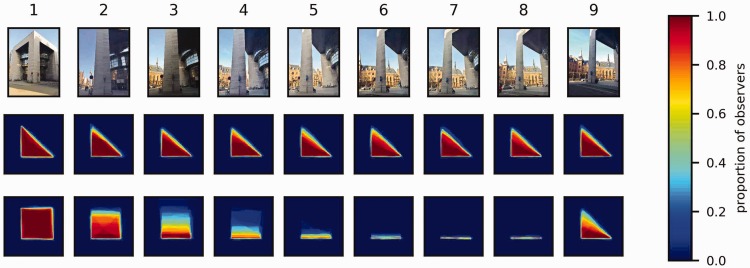
Aggregated representations of the drawings made by the observers according to the cognitive criterion (middle row) and according to the perceptual criterion (bottom row). The number above each panel indicates the viewing position (see Figure 5). The color of each pixel indicates the proportion *p* of the individual drawings for which this pixel was contained within (or at the boundary of) the shape drawn. Thus, dark blue pixels (*p* = 0) show regions that were never part of the shapes drawn by the observers, and dark red pixels (*p* = 1) show regions that were part of the all the individual drawings. Intermediate values represent lesser degrees of consistency across observers. A proportion of 0.5 represents the minimal possible consistency across observers.

Figure 8 shows representations of the shapes drawn aggregated across observers for each viewing position (columns) and task criterion (cognitive in the second row and perceptual in the third row). The photos in the first row show the observers’ view of the pillar at the given viewing position (Figure 5). At any given point in these plots, the pixel value indicates the proportion of the observers for which this point fell within (or at) the boundary of the shape they had drawn (see Appendix for details). Note that a pixel value of 0 or 1 indicates perfect agreement between all observers (the point is either outside the drawing for all observers or inside the drawing for all observers) and that a pixel value of 0.5 represents the minimal possible agreement (inside for half of the observers and outside for the other half).

In agreement with the qualitative shape analysis, the drawings made according to the *cognitive criterion* (middle row) agree quite well with the true shape of the pillar for all of the nine viewing positions and were also rather similar across observers. The drawings made according to the *perceptual criterion* (bottom row) are clearly different: First, the shapes drawn deviated considerably from the true shape at all viewing positions except number 9, where the deviation from the true shape is noticeable, but less dramatic. Second, the shapes drawn depend strongly on the viewing position. Third, the interobserver agreement seems to range from very high for some viewing positions (e.g., Viewing Position 1) to very low for other viewing positions (e.g., Viewing Position 3).

**Figure 9.**
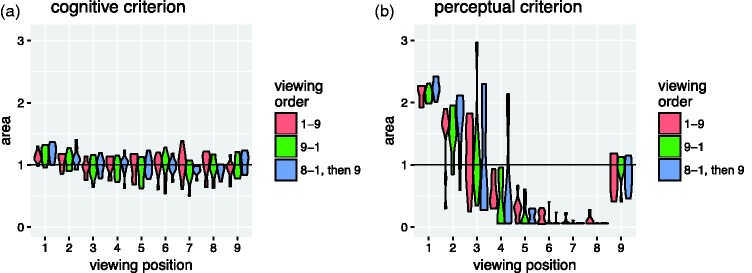
Violin plots of the area of the observers’ drawings. The area values are expressed relative to the true area of the pillar’s cross section (solid horizontal line). The estimated density distributions are clipped at the minima and maxima of the actual distributions. The areas of the drawings made according to the cognitive criterion (a) exhibit relatively little variability across observers and correspond roughly to the true area. For the drawings made according to the perceptual criterion (b), however, both the average areas and the variability across observers depend strongly on the viewing position. Note that the violin plots are scaled to the same width at the maxima of each distribution such that the actual densities are correspondingly greater for the narrower distributions.

**Figure 10.**
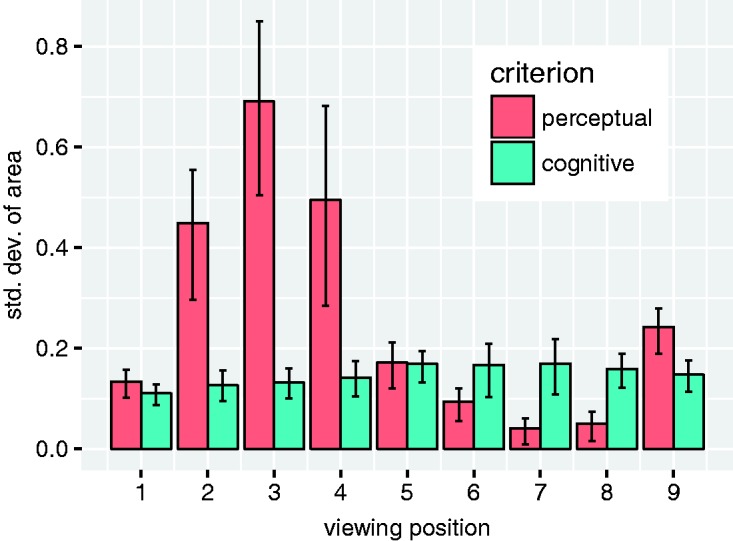
Standard deviation of the area of the observers’ drawings. The error bars represent 95% confidence intervals obtained through bootstrapping. While the interindividual variation is roughly constant across viewing positions for the drawing made according to the cognitive criterion, it depends strongly on viewing position for drawing made according to the perceptual criterion: It is particularly large at Viewing Positions 2 to 4 and particularly low at Viewing Positions 8 and 9.

Figure 9 illustrates how the areas of the individual observers’ drawings are distributed for each combination of viewing position, task criterion, and viewing order. Note that the violin plots are scaled to the same width at the maxima of each distribution such that the actual densities are correspondingly greater for the narrower distributions. As can be seen, the pattern of results is similar for each of the three conditions with different orders of presentation. The interobserver variability is roughly constant across viewing positions for the drawings made according to the *cognitive criterion*, but for those made according to the *perceptual criterion*, the interobserver variability depends strongly on the viewing position: It is particularly large at Viewing Positions 2, 3, and 4, and it is particularly small for Viewing Positions 6, 7, and 8. This is also evident in Figure 10, which shows the standard deviations of the area distributions with 95% confidence intervals obtained through bootstrapping.

## Discussion

The results of our experiment show that the very same triangular pillar can be perceived as having radically different 3D shapes depending on the position from which it is viewed: It can be perceived as a very thin plane or as having another rectangular, square, or triangular cross section. Importantly, the illusory perceptual experiences of a rectangular cross section persist despite explicit conscious knowledge of the true triangular cross section. That is, these perceptual experiences are cognitively impenetrable (Ekroll et al., 2013, 2016; Ekroll, Sayim, & Wagemans, 2017; Firestone & Scholl, 2015; Kanizsa, 1985; Leslie, 1988; Nielsen, 2008; Pylyshyn, 1999).

### Perceptual Preference for Rectangularity/Symmetry

At all viewing positions except Viewing Position 9, the overwhelming majority of the drawings made by the observers to indicate their perceptual impression of the pillar’s 3D shape were rectangular (in the general sense, including a thin line and a square, see Figure 7(c)). This finding suggests that the perceptual system relies on preference for rectangularity (Attneave & Frost, 1969; Griffiths & Zaidi, 2000; Halper, 1997; Kubovy, 1988; Tjan & Ruppertsberg, 2001; Thrun & Wegbreit, 2005) whenever the visible parts of the object are compatible with a rectangular interpretation. This is the case at all viewing positions except number 9, where the two short sides of the pillar forming a 45° corner are directly visible. An alternative interpretation would be that the perceptual system relies on a preference for symmetry (Attneave & Frost, 1969; Hochberg & McAlister, 1953; Koffka, 1936; Li, Pizlo, & Steinman, 2009; van der Helm, 2014, 2015; van Lier & Wagemans, 1999; Wagemans, 1995, 1997) rather than rectangularity per se. An advantage of this hypothesis is that it is more general and can be applied to objects with nonplanar surfaces. The observations that a semispherical shell is perceived as a complete ball (Ekroll et al., 2013, 2016), for instance, could be also be attributed to a tendency toward symmetry. Some aspects of our findings may, at first blush, appear to be at odds with the symmetry hypothesis. First, a square has two more mirror symmetries (the diagonals) than a nonsquare rectangle. Hence, one would expect the observers to report a square cross section at Viewing Positions 1, 2, and 3 (where the visual input is compatible with such an interpretation), but this is mostly not the case at Viewing Position 3 and only partially the case at Viewing Position 2 (see Figure 7). Second, the drawings made at Viewing Position 9 were often a bit flattened relative to the true isosceles right triangle (as evident in Figure 4 and indirectly via the area data in Figure 9(b)), which means that the observers tended to report a shape that is less symmetrical than the true shape, which has one plane of symmetry. It is conceivable, though, that these deviations from optimal symmetry can be attributed to other factors also playing a role, such as a tendency to minimize the surface area of an object (Li et al., 2009; Pizlo, Li, Sawada, & Steinman, 2014). Furthermore, one might ask why an equilateral triangle—which may be considered more symmetrical than a (nonsquare) rectangle because it has three axes of symmetry—hardly ever showed up in the participants drawings although it would be compatible with the single façade visible from many of the viewing positions (e.g., 2 and 3). One could argue, though, that if a square and an equilateral triangle were to be squashed along one dimension due to the previously mentioned tendency to minimize the surface area, the resulting nonsquare rectangle would have more symmetries than the resulting isosceles triangle.

A striking feature of the observer’s drawings made according to the perceptual criterion is that they consistently correspond to a very thin rectangle (or a line) at Viewing Positions 6 to 8, while the rectangles drawn are thicker and more variable across observers at Viewing Positions 2 to 5 (see Figures 7 and 8), although the directly visible part of the building (one short side) is the same at all these viewing positions. How can we explain these differences? A fundamental difference between Viewing Positions 2 and 3 on one hand and Viewing Positions 4 to 8 on the other hand is that the latter are located to the right of the rightmost point of the pillar (Figure 5). In these cases, the assumption that the base of the pillar is rectangular entails that the right-hand side of the rectangle must be visible, which, of course, it is not. Hence, at Viewing Positions 4 to 8, there is sufficient information in the visual input to conclude that if the cross section is rectangular, it can only be a thin line. This explanation accounts very well for the data on Viewing Positions 6 to 8 but not so well for the somewhat larger areas and individual differences at Positions 4 to 5.

It is also presently unclear to us why the areas tend to be larger at Viewing Position 2 than at Viewing Position 3. The most obvious difference between these to viewing positions is that Viewing Position 3 corresponds to the midpoint of the visible side, while Viewing Position 2 is located further to the side.

The preference for symmetry suggested by the present findings has already been anticipated by Tse (1999a, pp. 61–62) in his pioneering article on amodal volume completion. Here, he briefly and informally considered how we come to perceive a cubic building as such even though only two of its sides are visible, suggesting that “the bias to see volume in such cases is due to object knowledge or templates that inform our judgments about likely 3D form” and that there may “be an assumption that the back of an object is like the front.”

Both the notion that the back of the object is like the front and the informal general notion of symmetry are fairly broad concepts that can be formalized in different ways, leading to different predictions. For instance, two visible sides of unequal length at an obtuse angle could be completed into both a parallelogram and a kite shape, but which of these solutions is more symmetric? The kite shape has two mirror symmetries, and the parallelogram has no mirror symmetries, but the parallelogram is point-symmetric. With the particular stimulus used in the present study, many such distinctions are confounded (a square is a special case of both a kite and a parallelogram); hence, further work with other stimuli is needed to establish more concretely what kinds of symmetries the visual system prefers. While the stimuli obviously do not have to be buildings, it is probably important that they are tall enough such that the observers can see neither the bottom nor the top. As it might be impractical to search for or construct suitable objects or buildings, and pictures on a computer screen might be too impoverished to yield very clear percepts of amodal volume completion, it might be a good idea to use stimuli rendered in virtual reality.

The idea that a preference for symmetry drives amodal volume completion can, of course, only work, provided that the shape of the visible front is already known, and the perceptual inference of visible 3D shape is itself a nontrivial problem. Tse (2002; see also Albert & Tse, 2000; Tse, 1998; Tse & Albert, 1998) has proposed an approach to surface filling-in and volume formation according to which the visual system “may seek out ‘propagable’ segments of occluding contour that could project from segments of *rim* lying on a planar cut […] or cross-section of a volume” (p. 91). These propagable segments can then be “generalized over ambiguous portions of the image to generate the percept of a volume”. This general approach can be applied not only to volumes with everywhere differentiable surfaces but also to volumes that have corners, like the building we used in the present study. This approach does not in itself make explicit predictions about amodal completion of the back of the volume, but if it is supplemented by assumptions about how the propagable segments are completed behind the object, it certainly could.

### Distinct and Vague Perceptual Representations

As can be seen in Figures 9 and 10, the interindividual variability in the area of the drawings made according to the perceptual criterion is particularly high at Viewing Positions 2 to 4 and particularly low at Viewing Positions 6 to 8. Our own informal phenomenological observations indicate that the amodal shape percepts are particularly compelling and distinct at some viewing positions and less so at others. Hence, it seems plausible that the particularly high interobserver variability observed at Viewing Positions 2 to 4 results because the observers do not have a very clear and compelling perceptual impression to base their reports on in these cases. Conversely, the particularly low interobserver variability at Viewing Positions 6 to 8 agrees with our impression that the percept of a very thin cardboard-like wall is very crisp and distinct.

The high interobserver variability at Viewing Positions 2 to 4 is open to two interpretations. One possibility is that, while the observers do have perceptual representations of the pillar’s backside at the other viewing positions on which to base their judgments, this is not the case at these particular viewing positions—possibly due to a lack of whatever cues the perceptual system uses to create amodal representations. Thus, the observers are not really able to report on any perceptual impression and have to rely on pure guesswork instead, which could explain the high variability. An alternative possibility is that the observers also have perceptual representations at these viewing positions but that they are more abstract and fuzzy in nature. Given that the observers almost always draw shapes that can be described as rectangles in the broad sense (including a square), these abstract perceptual representations could be regarded as sets of rectangles with aspect ratio as a free parameter. This latter interpretation is supported by the observation that the shapes drawn by the observers at these viewing positions (according to the perceptual criterion) were neither arbitrary, nor in any way related to the observers’ knowledge of the true triangular shape of the pillar, but rather almost exclusively rectangles with different aspect ratios. Reliance on idiosyncratic and inflexible rules such as the ones suggested by this observation is typical of perceptual processing (Kanizsa, 1985).

**Figure 11.**
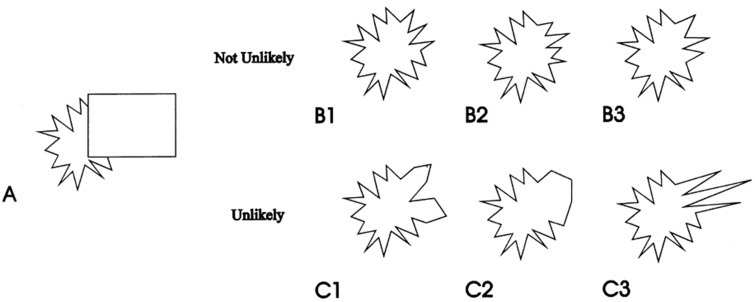
The shapes B1 to B3 and C1 to C3 are all logically possible completions of the partially occluded shape in A. Some of them (B1 to B3) are experienced as likely, while others (C1 to C3) are experienced as unlikely. Thus, the mental representation of the hidden parts of the shape may be better conceived of as a set of possible shapes than as a specific one. Adapted from “Investigating Global Effects in Visual Occlusion: From a Partly Occluded Square to the Back of a Tree-Trunk,” by R. van Lier, 1999, *Acta Psychologica*, *102*(2), pp. 203–220. Copyright (1999) by Elsevier. Reprinted with permission.

A possibly related abstract and fuzzy representation of occluded scene regions has been described by van Lier (1999). Most examples of amodal completion discussed in the literature refer to cases where the shape of the occluded part of an object is experienced as very distinct and specific, but Figure 11, which is reproduced from van Lier’s (1999) work, illustrates how our experience of the occluded part of an object may be quite abstract or fuzzy, yet at the same time not completely arbitrary (see Koenderink, 2015 for a related general point about the abstract nature of visual surfaces). The mental representation of the hidden parts of the jagged shape in Figure 11 may be better conceived of as a set of possible shapes than as a concrete, specific one. The notion of abstract and fuzzy representations in amodal perception has also been discussed in connection with the recently described phenomenon of amodal absence (Ekroll et al., 2017). It is also worth noting that fuzzy perceptual representations are well known and seem to play an important role in other domains of perception such as peripheral vision (Balas, Nakano, & Rosenholtz, 2009; Coates, Wagemans, & Sayim, 2017; Koenderink, Valsecchi, van Doorn, Wagemans, & Gegenfurtner, 2017; Sayim & Wagemans, 2017). Although the present observations highlight the role of perceptual mechanisms, gaining a better understanding of the relative roles and the interplay between perceptual and cognitive factors in creating our experience of occluded scene regions clearly remains an important goal for future research (Hazenberg, Jongsma, Koning, & van Lier, 2014; Hazenberg & van Lier, 2015; Vrins, de Wit, & van Lier, 2009).

### Smooth Continuation Versus Sharp Corners in Amodal Completion

Much previous work on amodal completion appeals to the Gestalt principle of good continuation (Wertheimer, 1923/2012) or various modern incarnations of it such as contour relatability Kellman and Shipley (1991), surface relatability, or good volume continuation/complete mergeability Tse (1999a, 1999b). In agreement with the basic intuition behind the principle of good continuation, there is much evidence that amodal completion tends to yield smooth percepts (e.g., Gerbino, 2017). The drawings made by the participants in the present study, however, suggest that the visible sides for the pillar evoked the experience of amodal volume completions that have sharp edges rather than smooth continuations (corners that are not directly visible). As such, our findings are in agreement with Tse’s (1999a) informal observation that two visible planar surfaces can give rise to the perception of “a cubic volume, like a building” (pp. 61–62) and the corollary that there is “more contributing to volume completion than surface relatability or volume mergeability” (p. 61). Given the relative sparsity of hard evidence for sharp corners in amodal completion relative to the large body of evidence for smooth continuations, however, one might still ask whether the present findings are the result of some kind of artifact.

In cases where the amodal percept is distinct and clear, we find it reasonable to assume that the “draw-what-you-experience” task represents the observers’ actual percepts reasonably well up to inaccuracies that may result simply because our observers were not trained artists. In cases where the amodal percept is indistinct, fuzzy, or even absent altogether, however, it is logically impossible for the observers to make a simple line drawing that accurately represents their perceptual experience. As discussed earlier, the amodal percept seems to be very distinct and compelling at Viewing Positions 6 to 8, both in light of the low interobserver variability and our own informal phenomenal observations, so in these cases, we are fairly confident that the drawings represent the perceptual experience adequately. At Viewing Positions 2 to 4, however, the amodal percept seems to be much less well defined or perhaps even absent altogether. In these cases, the task of drawing a single outline that represents the perceptual experience may be downright impossible. Faced with such an impossible task, the observers may have been particularly susceptible to subtle unintended demand characteristics that may have biased their responses. For instance, the initial demonstrations with the shell-sphere effect may have biased the observers to make drawings that were also symmetrical. Because the agreement between observers was very high at Viewing Position 1, the results obtained here provide perhaps the strongest evidence for sharp corners in the amodal percepts. But it must be admitted that the high interobserver agreement does not necessarily imply that the percept is distinct and compelling. Our own informal phenomenological observations suggest that it is more distinct and compelling than what one experiences at Viewing Positions 2 to 4 but less distinct than what one experiences at Viewing Positions 6 to 8. This issue could be resolved in future work by using tasks that also provide information about the phenomenal distinctness/fuzziness of the observers’ perceptual experiences.

### Breakdown of Amodal Shape Constancy

Apart from a few scattered early discussions (Johansen, 1957; Michotte & Burke, 1951), the phenomenon of amodal volume completion was largely neglected for a long time in vision science until more recent work (Tse, 1999a; van Lier, 1999; van Lier & Wagemans, 1999) made its existence and importance for perception theory more readily apparent. One reason why it is easy to overlook the pervasive role of amodal volume completion *qua* perceptual phenomenon in everyday perception is that our perceptual impressions of the backsides of things tend to agree with our explicit knowledge of the 3D shape of things. Our perceptual impressions of the backsides of things only become readily apparent when they are at odds with our explicit knowledge. The dramatic lack of perceptual 3D shape constancy across different views of a triangular pillar demonstrated in the present study is particularly illustrative in this regard. Three-dimensional shape constancy is known to be very good in a great many cases (Pizlo, 2010; Pizlo et al., 2014), but it is also known to break down in some cases (Rock & DiVita, 1987; Rock, Wheeler, & Tudor, 1989). Such breakdowns may be particularly useful in inferring the general rules underlying 3D shape perception. One might speculate that the perceptual preference for rectangularity/symmetry becomes particularly evident in the present study precisely because other cues and information that normally also constrain the perceptual inference of 3D shape are largely absent in this situation.

The illusion described in the present study might not be very frequent in natural circumstances, although after we became aware of it, we have observed it in a great number of buildings with corners forming an acute angle. Obviously, the illusions would not occur if the pillar were much shorter or oriented differently such that the “top” or “bottom” were visible to the observer.

### Amodal Perception of Empty Space

According to our own informal observations, the illusion that the pillar looks flat and thin like cardboard is also accompanied by a rather enigmatic but quite forceful impression or “feeling” that the space behind the thin wall is empty, although, of course, the space behind the wall is not directly visible. Note that this impression, which we find very compelling when viewing the actual pillar, is much reduced or even entirely lost in photographs of it, presumably because the sense of “immersive negative space,” which is one of the major phenomenological characteristics of stereopsis (Vishwanath, 2014, p. 153), is lost in the photograph. Similar observations regarding the perception of absence (Farennikova, 2013) or “amodal absence” (Ekroll et al., 2017) have recently been made, and this enigmatic phenomenon invites further study.

### The Role of Amodal Completion Based on Symmetry/Rectangularity in Magic

Ekroll et al. (2017) have recently argued that amodal perception is a major factor in a wide range of magic tricks. The results of the present study points to the role of a perceptual preference for rectangularity or symmetry in amodal volume perception, and it might be instructive to briefly consider a few examples of how these principles are applied in magic.

**Figure 12.**
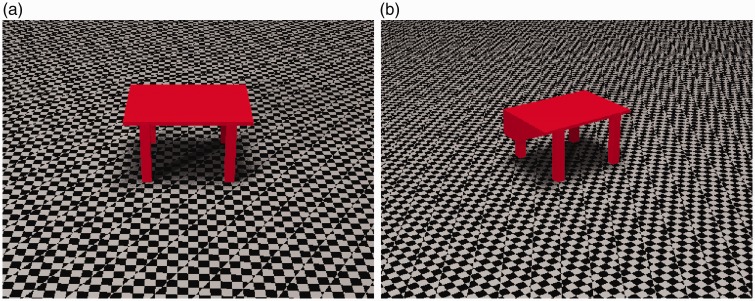
Two views of the same table. When viewed from the front (a), the table looks like a single thin surface, but as shown in (b), the table is actually getting thicker toward the back and has plenty of space for hiding things.

Sharpe (1992, p. 16) describes how magicians use tables with a tabletop that becomes thicker toward the back, thereby creating the illusion that the tabletop is quite thin although there is plenty of space to hide things in the back part of it (see Figure 12). The results of the present study suggest that this illusion is perceptual in nature, which would explain why it is very useful to magicians, and that the underlying principle is a preference for rectangularity or symmetry.

**Figure 13.**
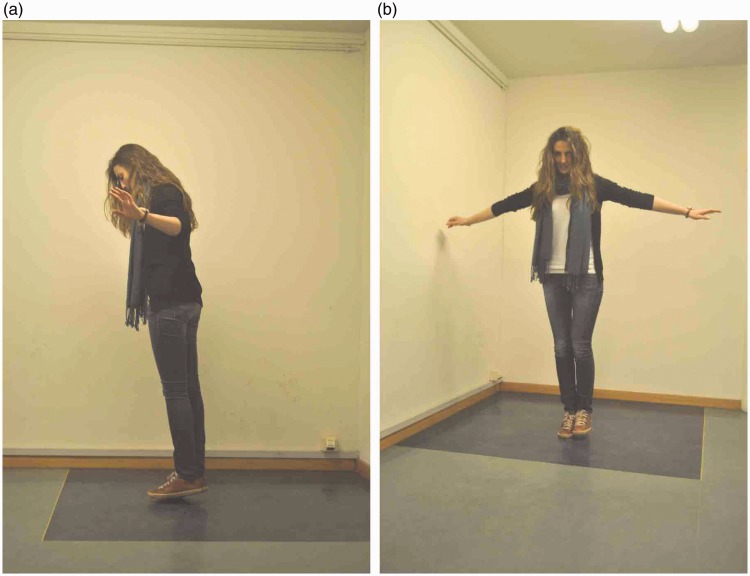
Balducci levitation. (a) The magician creates the illusion of levitating a few inches above the ground by (b) balancing on the toes of her hidden foot.

A well-known magic trick known as Balducci levitation^3^ can also be explained by appealing to amodal volume completion based on a preference for symmetry. As shown in Figure 13, the magician creates the compelling illusion of hovering a few inches above the ground by standing on the toes of the foot that is hidden from the spectator’s view. In light of the present findings, it appears likely that the illusion of floating occurs because the spectator’s perceptual system creates an amodal representation of the hidden foot that is symmetrical to the visible foot. An additional factor that may play a role in this illusion is the amodal perception of empty space discussed earlier (Ekroll et al., 2017): Logically, floating in thin air entails empty space behind/below the feet, and the perceptual impression of empty space may be important in creating the illusion of floating. It would seem that this illusion is more compelling in a real scene than on a photograph, presumably for the reasons already discussed in the earlier section.

A method playing a central role in a card trick where a card selected and remembered by the spectator is seemingly inserted back into the middle of the deck, but immediately afterward appears at the top of the deck is known as Marlo’s tilt.^4^ While it looks like the card is being put into the middle of the deck, it is actually put in below the topmost card, and by performing a so-called double-lift (turning the two top cards together as if they were one card), the magician makes it appear on top. The illusion that the card is inserted in the middle of the deck is created by tilting all but the top card downward while keeping them flush with the top card at the front visible to the spectator. The illusion that all the cards in the deck together form a symmetrical/rectangular object although they actually form a triangular object with a large gap in the back makes it appear that the card is being inserted into the middle of the deck rather than as the second card below the top card.

## Conclusions

The results of our experiment reveal striking examples of amodal volume completion, where observers have compelling and cognitively impenetrable perceptual experiences of the hidden backside of an object. They also provide strong evidence that the visual system uses a preference for rectangularity or symmetry to determine the 3D shape of objects.

## Notes

1 We did not measure the height of the pillar, but it is probably more than 10 m high.

2 The location of the pillar is Andreas Vesaliusstraat 13, 3000 Leuven.

3 See https://www.youtube.com/watch?v=TaLdMkAmIsg, link (last accessed on May 14, 2018).

4 See https://www.youtube.com/watch?v=h1rL8zsMg-w, link (last accessed on May 14, 2018).

## Appendix

To indicate their experience of the pillar’s 3D shape, the observers drew horizontal cross sections in red on top of a map indicating their own location and the visible short sides of the pillar. The completed drawings (examples shown in Figure 6) were saved as raw bitmaps (960 × 720 pixels). Because the drawings made by the observers fell within a limited region of these raw bitmaps, further processing was based on a smaller square region of the raw bitmaps measuring 200 × 200 pixels. We separated the observers’ drawings from the predrawn details of the maps by coding the red pixels in the raw bitmaps as 1 and all other pixels as 0. Next, we converted the outline shapes to filled shapes using the SciPy function “ndimage.morphology.binary_fill_holes.” In a few cases, the observers had failed to draw a completely closed contour, leaving a small gap between the beginning and the end of the trace drawn. Because the filling algorithm requires a closed contour as input, we manually closed the small gaps in the contour in these cases. The middle and bottom panels in Figure 8 were obtained by superimposing these bitmaps across all observers and dividing the resulting pixel value by the number of observers. The area values were obtained by counting the number of pixels in the filled drawing and dividing by the number of pixels for a corresponding bitmap of the true triangular shape.
